# Tight docking of membranes before fusion represents a metastable state with unique properties

**DOI:** 10.1038/s41467-021-23722-8

**Published:** 2021-06-14

**Authors:** Agata Witkowska, Leonard P. Heinz, Helmut Grubmüller, Reinhard Jahn

**Affiliations:** 1 Laboratory of Neurobiology, Max-Planck-Institute for Biophysical Chemistry, Göttingen, Germany; 2 Department of Theoretical and Computational Biophysics, Max-Planck-Institute for Biophysical Chemistry, Göttingen, Germany; 3 University of Göttingen, Göttingen, Germany; 4 Present Address: Department of Molecular Pharmacology & Cell Biology, Leibniz-Forschungsinstitut für Molekulare Pharmakologie (FMP), Berlin, Germany

**Keywords:** Membrane biophysics, Membrane structure and assembly

## Abstract

Membrane fusion is fundamental to biological processes as diverse as membrane trafficking or viral infection. Proteins catalyzing membrane fusion need to overcome energy barriers to induce intermediate steps in which the integrity of bilayers is lost. Here, we investigate the structural features of tightly docked intermediates preceding hemifusion. Using lipid vesicles in which progression to hemifusion is arrested, we show that the metastable intermediate does not require but is enhanced by divalent cations and is characterized by the absence of proteins and local membrane thickening. Molecular dynamics simulations reveal that thickening is due to profound lipid rearrangements induced by dehydration of the membrane surface.

## Abstract (web summary)

Proteins need to overcome energy barriers to induce intermediate steps in membrane fusion. Using lipid vesicles in which progression to hemifusion is arrested, the authors show that the metastable intermediate is enhanced by divalent cations and is characterized by the absence of proteins and local membrane thickening. Simulations reveal that thickening is induced by dehydration of the membrane surface.

## Introduction

Fusion of biological membranes is fundamental for the functioning of all living organisms ranging from the cell entry of enveloped viruses to the exocytotic release of neurotransmitters. While the proteins mediating fusion are evolutionarily unrelated and structurally diverse, the merger of two bilayers appears to follow a common pathway involving a sequence of structurally distinct intermediates. These begin by loose protein-mediated membrane contact and is followed by tight apposition of the membranes while still maintaining bilayer integrity. Then, membrane structure is disrupted by the merger of the proximal leaflets, resulting in a fusion stalk or a hemifusion diaphragm. This is followed by the rupture of the diaphragm (fusion pore) that then expands, thus completing membrane merger^1,2^.

While the pathway outlined above is supported by an increasing body of experimental evidence and theoretical modeling^1–5^, there are still crucial gaps in knowledge, in particular with respect to the steps immediately before the first bilayer disruption. Importantly, these are also the steps on which proteins regulating final progression to fusion operate. Thus, we need to clarify the structural and energetic details of these intermediates to precisely understand the molecular mechanisms of such regulatory proteins (e.g., synaptotagmins) that are controversially discussed since more than 20 years^5–7^.

Here we focus on the tightly docked intermediate in which membranes are apposed to each other with a distance of <1 nm. While this state is well known from cryo-electron microscopy (cryoEM) studies in events as diverse as fusion of trafficking organelles^8–10^, fusion of mitochondria^11^, or cell entry of influenza virus^12^, its biophysical features have remained largely enigmatic. To reach such state, repulsive forces between negatively charged lipid headgroups and the energy barrier involved in dehydration need to be overcome. It is unclear, which forces are counteracting (headgroup chelation by divalent cations, van der Waals and hydrophobic forces, protein “clamps”), if and how proteins are cleared from these contact sites, and how the subsequent transition to stalk formation is facilitated^1,2,13^.

**Fig. 1:**
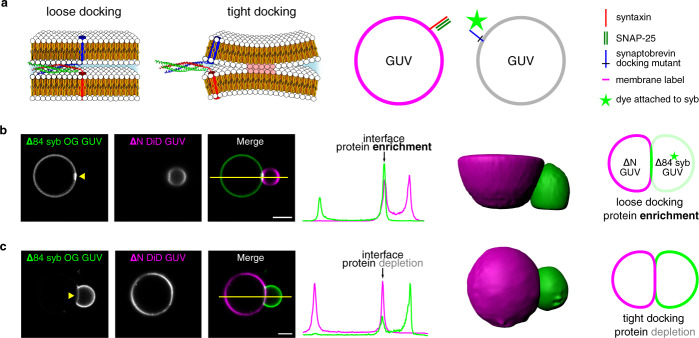
Distribution of SNARE proteins at the docking interface differs between loose and tight docking states. **a** Schematic illustration showing loose and tight docking states (left) and the experimental system used in the docking experiments (right). One set of GUVs contain the lipidic membrane dye DiD (magenta) and unlabeled Q-SNARE complexes, whereas the other set of GUVs contains the synaptobrevin docking mutant ∆84 syb, labeled with Oregon Green 488 (OG, green). SNARE complex structure PDB ID: 3IPD^39^. **b**, **c** Examples (from >5 independent experiments) of a loosely (**b**) and tightly (**c**) docked GUV pair, respectively, imaged by fluorescence microscopy. The position of a line scan for fluorescence intensities (graph next to images) is marked with a yellow line. Labeled synaptobrevin is enriched at the interface in the loosely docked state and depleted at the interface in the tightly docked state (see also schematic drawing on the right). Scale bars 5 μm. Source data are provided as a Source Data file.

Here we have used an in vitro system in which fusion of artificial membranes is mediated by SNARE (soluble N-ethylmaleimide-sensitive-factor attachment receptor) proteins involved in neuronal exocytosis. SNARE proteins contain conserved stretches of 60–70 residues belonging to four subfamilies, termed Qa-, Qb-, Qc-, and R-SNARE motifs. These proteins are known to fuse membranes by a consecutive assembly of complementary SNAREs (one SNARE motif of each subfamily) that is initiated at the N-terminal ends and progresses towards the C-terminal membrane anchors, referred to as SNARE zippering^5,6^. The in vitro system was optimized for studying fusion intermediates by omitting all upstream steps regulating initial assembly^14^ and consists of an activated Q-SNARE complex in the one membrane and the R-SNARE in the other membrane. We have shown previously that in this system the tightly docked state can be stably reproduced as a fusion intermediate and furthermore, that it can be arrested in this state by a point mutation in the R-SNARE synaptobrevin^9,15^. As shown in Fig. 1a (right), membrane contact results in N-terminal assembly of the SNARE proteins. In this state (referred to as loosely docked state), membranes are still separated by a hydrated gap, and disruption of SNARE assembly by the disassembly through ATPase NSF or by competition with soluble synaptobrevin fragments leads to vesicle dissociation^9^. The system then progresses to the tightly docked state that cannot be reverted anymore by SNARE disassembly suggesting the involvement of attractive forces of unknown nature that only operate at subnanometer distances.

## Results

### Localization of SNARE proteins at membrane docking sites

To allow for using light microscopic techniques for the characterization of the intermediates, we adapted this system by using GUVs (giant unilamellar vesicles) instead of large unilamellar vesicles. Two sets of GUVs were reconstituted with complementary sets of SNARE proteins—synaptobrevin-2 (syb) that in neurons resides on synaptic vesicles and a stabilized complex containing syntaxin-1A and SNAP25a^14^ that are naturally present on the plasma membrane. In this system, syb contains a single residue deletion (∆84) that is known to trap fusing membranes at the tightly docked state^8,9,15^. In one vesicle set, membrane of a GUV was labeled allowing to control localization of lipids as well as making sure that no hemifusion has occurred. In the other set of vesicles, syb was labeled to allow for monitoring of protein behavior at the interface (Fig. 1a left). When mixed together, these vesicles would interact with each other, fuse, or get stalled at one of the fusion intermediates such as docking or hemifusion. The low curvature of the GUV membrane leads to a higher energy barrier for membrane merger^8^, thereby increasing the probability of obtaining arrested docked states.

**Fig. 2:**
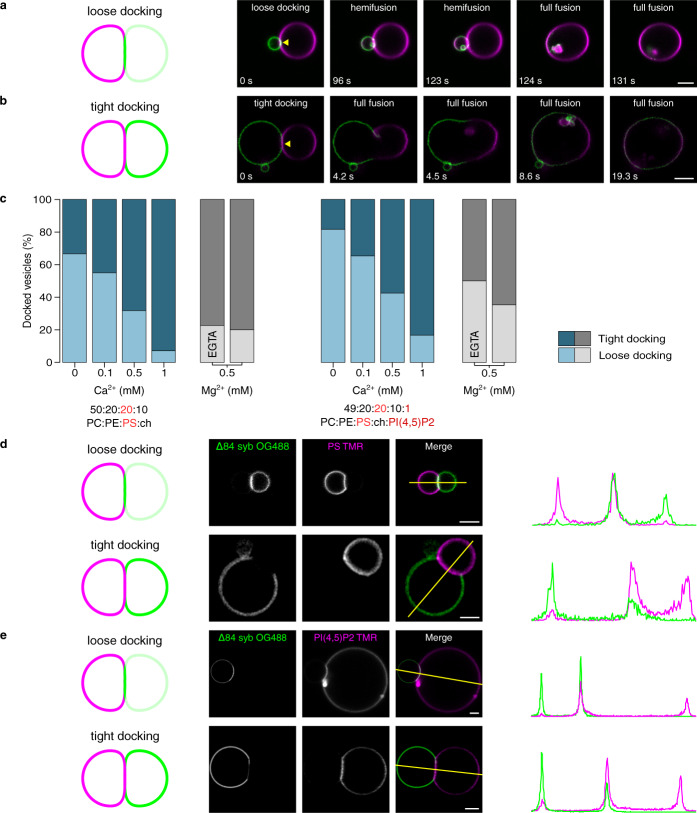
Loose and tight docking states are fusion intermediates enhanced by, but not dependent on divalent cations. **a,** **b** Time series of GUVs docked in loose (**a**) or tight (**b**) way transiting to the fully fused state (rare cases). Membrane and protein labeling as in Fig. 1. **c** Occurrence frequency of loose and tight docking in various concentrations of either Ca^2+^ or Mg^2+^ and in the presence of membranes that contain various amounts of the negatively charged lipids PS and PI(4,5)P2. Total number of interfaces used (bars from left to right): 42, 20, 44, 28, 22, 20, 49, 49, 40, 30, 18, 17. **d**, **e** Examples (from 3 independent experiments) of loosely and tightly docked GUV pairs containing ∆84 syb labeled with OG (green) and membranes containing TMR labeled PS (**d**) or PI(4,5)P2 (**e**) (purple). PS distribution remains unaffected in both docking types while there is an enrichment of PI(4,5)P2 at loose docking interfaces. Scale bars 5 μm. Source data are provided as a Source Data file.

If, as we suggested previously, SNAREs are excluded from tight docking interfaces, the protein density is expected to decrease in the membrane contact area. Conversely, at arrested loose docking interfaces, fusion complexes in *trans* will get trapped and thus accumulate over time, visible as increased protein signal at the interface. Indeed, with our experimental system we were able to demonstrate two distinct docking states preceding hemifusion, which are characterized by either protein accumulation at the docking interface (loose docking, Fig. 1b) or protein depletion from the membrane-membrane contact site (tight docking, Fig. 1c). Moreover, in some rare cases, we were also able to observe transition of these trapped intermediates to full fusion as shown in Fig. 2a, b (see also Supplementary Movies S1–S2) confirming that the tightly docked state is an intermediate step in the fusion pathway.

### Divalent cations regulate tight docking state formation

To better understand the forces that initiate and stabilize the tightly docked state we examined to which extent the formation of loose and tight docking interfaces, respectively, depends on the presence of divalent cations (Ca^2+^ or Mg^2+^). While increasing concentrations of both Ca^2+^ and Mg^2+^ augment the frequency of tightly docked intermediates, these intermediates also form in their absence (Fig. 2c). Intriguingly, very little difference between Ca^2+^ and Mg^2+^ was observed. Moreover, inclusion of the negatively charged lipid phosphatidylinositol 4,5-bisphosphate (PI(4,5)P2) in one of the docking membranes (as present on the plasma membrane) moderately reduces the frequency of tight docking interfaces indicating a somewhat higher energy barrier due to electrostatic repulsion (Fig. 2c right). Using fluorescently labeled variants of the acidic membrane lipids (PS-TMR or PI(4,5)P2-TMR) we found that these lipid species are not depleted from the docking interfaces (Fig. 2d, e). Rather, a slight enrichment of PI(4,5)P2 was observed in the loose docking state (Fig. 2e), probably due to its binding to SNARE proteins (specifically syntaxin-1) accumulating at the interface. Together, these data show that tight docking is stable even in the absence of divalent cations despite the presence of acidic membrane lipids.

### Altered membrane thickness at tight docking interfaces

**Fig. 3:**
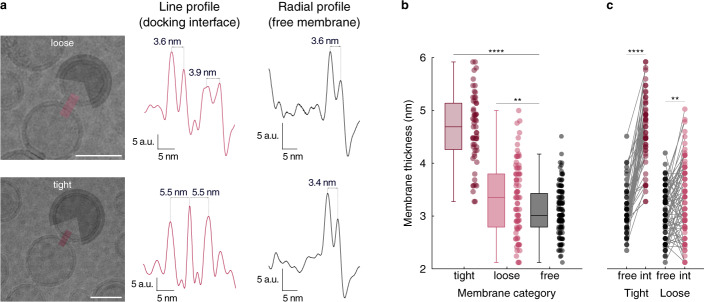
Membrane thickening at tight docking interfaces. **a** Membrane thickness of loosely (top) and tightly (bottom) docked interfaces, measured by line scans (at the interfaces) and radial profile analysis (membranes outside docking interfaces). Thickness is determined as distance between peaks corresponding to lipid headgroups between the two leaflets. Scale bars 50 nm. **b** Comparison of membrane thicknesses at tight (*n* = 50), loose (*n* = 72), and docking-free interfaces (*n* = 122) obtained by analyzing cryoEM images as shown in a. Boxes represent interquartile range, and whiskers below and above indicate full data range. Line in a box represents median. Data were analyzed with a one-tailed unpaired *t*-test at α = 0.05, ****P < 0.0001; **P = 0.0015. **c** Membrane thickness variations within single membranes being involved in docking interfaces (int) or free (*n*_Tight_ = 50; *n*_Loose_ = 72). Data were analyzed with the Wilcoxon matched-pairs signed-ranks test at *α* = 0.05, ****P < 0.0001; **P = 0.006. Source data are provided as a Source Data file.

Previously^9^ different membrane-membrane distances were observed between docked vesicles by cryoEM. Specifically, in tightly docked membranes with extended, flat docking interfaces (up to 100 nm-long for LUVs^9^) signals coming from lipid headgroups of proximal leaflets would blend together into one, thin line (see also Fig. 3a). We wanted to further characterize structural rearrangements of the membranes trapped in the tightly docked state and analyzed membrane thicknesses at docking interfaces in comparison to free membranes (Fig. 3a–c). Strikingly, we observed profound membrane thickening localized directly at the tight docking interfaces (Fig. 3). These structural changes were absent from free membranes and were also not observed at the interface of vesicle pairs classified as loosely docked (see Supplementary Information and Supplementary Fig. 1 for more details on the thickness measurements).

### Increased bilayer thickness is caused by surface dehydration

**Fig. 4:**
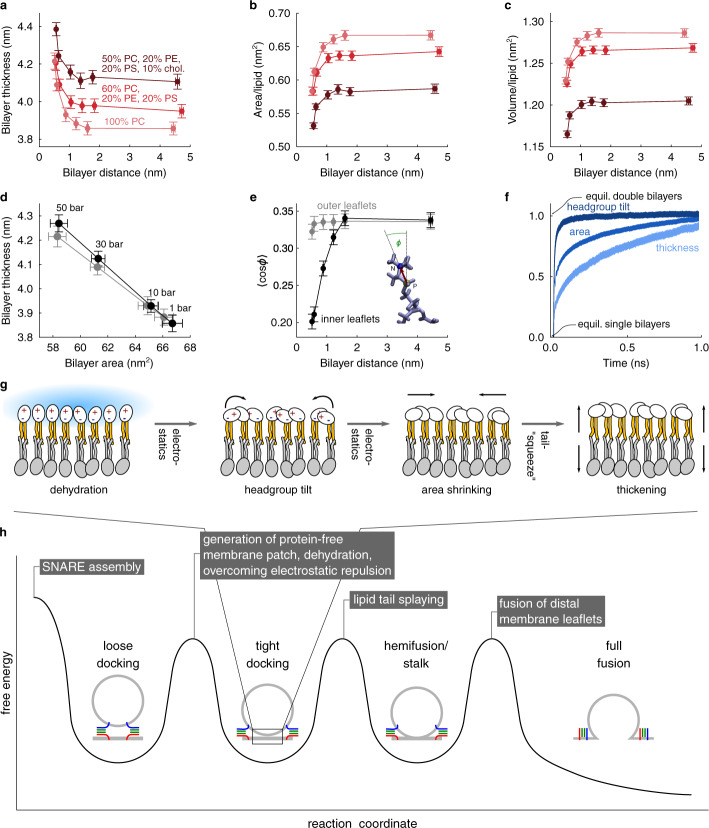
Membrane thickening at close distances is caused by dehydration-driven electrostatically-induced headgroup tilt and area compression. Atomistic molecular dynamics simulations show changes in membrane thicknesses (**a**), area (**b**), and volume (**c**) when two membranes with three different lipid compositions are apposed to each other at various distances. **d** Enforced membrane area compression reproduces membrane thickening at close distances. **e** Lipid headgroups within the inner membrane leaflet at the docking interface exhibit higher tilt (cos*ϕ*) upon close approach. Data points (**a**–**e**) are averages from 19000 points (190 ns) ± SD as described in *Methods*. **f** Change of headgroup tilt, area, and thickness over time during equilibration to a double-membrane stack (average over all 500 simulation runs; for easier comparison, all values were normalized to a range between 0 and 1). Accordingly, a value of 0 corresponds to equilibrated single bilayers and a value of 1 corresponds to equilibrated double bilayers. Data ± SD. **g** Scheme proposing order of events resulting in membrane thickening. **h** Cartoon showing a simplified energy landscape for membrane fusion. The energy barriers and minima are defined by observations of arrested intermediate states, their magnitude being arbitrary. Scaling on the *y*-axis is arbitrary. Source data are provided as a Source Data file.

To confirm that membrane thickening at close distances is due to membrane intrinsic properties, and to reveal the underlying molecular mechanism, we performed unbiased atomistic simulations of two opposing membranes at varying distances (Supplementary Fig. 2a, Supplementary Movie 3), mimicking the experimental conditions but in the absence of proteins. Indeed, for distances below 1.5 nm, significant thickening of the membranes was observed in the simulations, independent of the lipid composition (Fig. 4a and Supplementary Fig. 3). We also observed shrinkage of the membrane area (Fig. 4b), which raises the question which of the two changes is the primary cause. To answer this question, Fig. 4c shows that the volume of the membrane decreases with the membrane distance. Because the membrane behaves as a nearly incompressible fluid, this finding suggests that area shrinkage drives membrane thickening, as otherwise the volume should increase. Indeed, inducing a similar area shrinkage by increasing the lateral membrane pressure in additional simulations (Fig. 4d, black) quantitatively reproduces the thickening (gray).

Our simulations also showed that with decreasing distance, lipid chain order (Supplementary Fig. 2b) as well as headgroup tilt of the opposing membrane leaflets increase (Fig. 4e, black), whereas the outwards facing leaflets (Fig. 4e, gray) are nearly unaffected (see Supplementary Movie 4). The strong electrostatic dipole moment of the lipid headgroups suggests^16^ electrostatic interactions as the primary cause. In particular, the larger tilt of the lipid headgroups should allow for an electrostatically more favorable antiparallel arrangement. Indeed, the electrostatic interaction energy within the atoms of the inner leaflets decrease upon approach, whereas electrostatic interactions within the outer leaflets show only little change (Supplementary Fig. 2c).

To confirm that lipid headgroup tilting is the actual cause of area shrinkage, we enforced them to tilt in further simulations, which indeed reduced the membrane area (Supplementary Fig. 2d). We attribute these electrostatic effects to reduced shielding caused by dehydration, which was also seen in our simulations (Supplementary Fig. 2c). Independent support is provided by the timing of events, which follows the proposed causal chain (Fig. 4f and Supplementary Fig. 2e, f). Indeed, lipid headgroup tilting precedes area shrinkage, which precedes membrane thickening. Figure 4g summarizes the causal chain of events revealed by our atomistic simulations: Due to reduced electrostatic shielding, dehydration of the inner leaflet surfaces tilts the lipid headgroups, which laterally contracts the membrane. This contraction causes increases lipid ordering and, ultimately, drives thickening of the membrane.

## Discussion

Taken together, we now provide a coherent description of a metastable docking state that constitutes an intermediate with unique properties in the pathway leading to membrane fusion. Despite growing experimental evidence, it has so far been difficult to reconcile such a tightly docked intermediate with standard concepts in membrane biophysics. Current fusion models agree on the notion that due to electrostatic repulsion membrane contact destabilizes the membranes before the formation of a fusion stalk^2^. To minimize the energetic penalty of close membrane contact, many models imply that the contact area is limited by the formation of point-like membrane protrusions with fusion being facilitated by lipid packing defects at the apex of a highly deformed membrane^4^. Accordingly, tightly docked membrane-membrane interfaces reported in cryoEM studies^8–10^ were considered to be stalled, off-pathway states.

Our results significantly contribute to our understanding of the energy landscape governing the early steps of membrane fusion (Fig. 4h). After exergonic assembly of SNARE proteins^17^ a metastable state is reached where the membranes are still several nm apart from each other. This is followed by an energy barrier (Fig. 4h): Water and ions must be removed from the membrane contact zone, and the electrostatic repulsion between opposite lipid headgroups needs to be overcome^1,2^, possibly further assisted by membrane stretching generated by SNARE complexes. We now demonstrate that a metastable state follows that is characterized by a partially dehydrated and tight adhesion between membranes. The contact zone is free of proteins, does not require, albeit being stabilized by, divalent cations, and is associated with dehydration and a change in lipid organization, resulting in membrane thickening. Progression towards stalk formation probably requires tail splaying of membrane lipids^3^ which may be facilitated by the increased hydrophobicity and the changed lipid head geometry at the contact site. Probably, stalks are initiated at the edge of the membrane contact zone where increased curvature may cause lipid packing defects facilitating such transitions and assisted by assembly of SNARE complexes located at the rim^8^. Furthermore, we propose that in fast secretory cells such as neurons fusion may be arrested at this state. It is conceivable that the energy barrier separating this state from stalk formation and subsequent fusion is moderate (since energy barrier due to water and ions between membranes is not present any more) and can easily be overcome by accessory proteins perturbing lipid packing at the membrane surface, such as synaptotagmins.

## Methods

### Materials

Lipids: DOPC (1,2-dioleoyl-sn-glycero-3-phosphocholine), DOPE (1,2-dioleoyl-sn-glycero-3-phosphoethanolamine), DOPS (1,2-dioleoyl-sn-glycero-3-phospho-L-serine), 18:1 biotinyl cap PE (1,2-dioleoyl-sn-glycero-3-phosphoethanolamine-N-(cap biotinyl)), DOPS (1,2-dioleoyl-sn-glycero-3-phospho-L-serine), cholesterol (ovine wool), PI(4,5)P2 (L-α-phosphatidylinositol-4,5-bisphosphate, brain, porcine), TopFluor TMR (1-oleoyl-2-(6-((4,4-difluoro-1,3-dimethyl-5-(4-methoxyphenyl)-4-bora-3a,4a-diaza-s-indacene-2-propionyl)amino)hexanoyl)-sn-glycero-3-) PI(4,5)P2 and PS were purchased from Avanti Polar Lipids. Lipophilic tracer DiD, NeutrAvidin, biotinylated bovine serum albumin, were from Thermo Fisher Scientific.

### Protein expression, purification, and labeling

SNARE proteins (syntaxin-1A (183–288)^18^, SNAP-25 (cysteine free)^19^, synaptobrevin-2 Δ84 C28^20^, and synaptobrevin-2 fragment (49–96)^14^ were derived from *Rattus norvegicus*. Proteins were expressed in *Escherichia coli* strain BL21 (DE3) and purified via nickel-nitrilotriacetic acid affinity chromatography (Qiagen) followed by ion exchange chromatography on an Äkta system (GE Healthcare)^9,21^. The activated Q-SNARE complex^14^ consisting of syntaxin, SNAP-25, and synaptobrevin fragment 49–96 was obtained by overnight mixing at 4 °C in a buffer containing CHAPS, followed by ion exchange chromatography (MonoQ)^14,21^. Fluorescence labeling of synaptobrevin was carried out according to the manufacturer’s instructions using Oregon Green 488 (OG) iodoacetamide (Molecular Probes)^9^.

### Liposome and GUV preparation

For basic liposome mixtures the phospholipids PC, PE, PS, and cholesterol were mixed in a ratio of 5:2:2:1, respectively. In GUVs 1 mol% of DOPE was replaced with biotinyl-cap-PE for immobilization on neutravidin-functionalized coverslips^21^, and for fluorescent labeling 1 mol% of DOPC was replaced with the lipophilic tracer DiD. For localizing PS and PI(4,5)P2, 1 mol% was replaced with TMR-labeled versions of this lipids. Giant unilamellar vesicles were prepared by electroformation from small proteoliposomes (diameter ∼40 nm) containing either activated Q-SNARE complex (protein:lipid molar ratio 1:1000) or synaptobrevin (protein:lipid molar 1:500)^21^.

### Microscopy imaging and analysis

Imaging was performed on scanning confocal microscopes Zeiss LSM 780 and 880. Classification of docked vesicles was performed on docked GUV pairs (recognized based on labels in each of the GUV types). Pairs were classified as loosely docked if there was physical contact between vesicles, and if the fluorescent signal contributed by syb labeled with OG was not reduced in comparison to protein signal on the free membrane of syb vesicle at the interface. Pairs were classified as tightly docked if the syb signal was decreased or absent at the GUV-GUV docking interface. Enrichment or decrease of the syb signal at the interface was measured by line scans perpendicular to docking interface. Line scans and images for some visualizations were rotated using bicubic extrapolation rotation from Fiji^22^. 3D reconstructions were prepared with Imaris (Bitplane).

### CryoEM image analysis

Docked liposome pairs identified in cryoEM images with nominal magnification of 20,000–40,000× were analyzed with a custom written script executed in Fiji^22^ and GNU Octave^23^. Briefly, membrane thickness was extracted from line (docking interfaces) or radial (free membrane) profiles as distance between peaks denoting headgroups of both membrane leaflets. Radial profiles were obtained with the “Radial Profile Extended” plugin for ImageJ. Scripts used for this analysis are available on Zenodo^24^ (10.5281/zenodo.4642359).

### Molecular dynamics simulations

All molecular dynamics simulations were carried out using the software package Gromacs 2018^25–28^ with the CHARMM36m force field^29,30^. SETTLE^31^ and LINCS^32^ constraints were applied to all water molecules and bonds to hydrogen atoms respectively, and the system was time-propagated using the leap frog integrator with a time step of 2 fs. Electrostatic forces were calculated using the Particle-Mesh Ewald (PME) method^33^ with a 1.2 nm real space cut-off; the same cut-off was used for Lennard-Jones potentials^34^. The V-rescale thermostat^35^ with a time constant of 0.1 ps was used. Unless stated otherwise, all simulations were performed at 1 bar pressure, independently applied along the membrane and the lateral directions (semi-isotropic pressure coupling). Equilibration simulations were carried out using the Berendsen barostat with a time constant of 5.0 ps and production runs were performed using the Parrinello-Rahman barostat^36,37^ with a time constant of 1.0 ps.

For each of the tested lipid compositions, a symmetric bilayer of 100 lipids per leaflet was created using the MemBuilder II^38^ webserver (http://bioinf.modares.ac.ir/software/mb2/builder.php). Subsequently the systems were solvated using the Gromacs tool gmx solvate with approximately 18000 water molecules; wherever necessary, charge-neutrality was achieved by adding K-ions, and further ions equivalent to 75 mmol L^−1^ KCl were added, mirroring the experimental conditions. Gradient descent energy minimization was performed until a maximum force of 100 kJ mol^−1^nm^−1^ was reached, and the systems were equilibrated for 50.5 ns.

The equilibrated bilayers were duplicated to obtain double-membrane systems with inter-membrane distances varying between 0.4 and 4.7 nm, as shown in Supplementary Fig. 2a, with box sizes of approximately 8.1 nm × 8.1 nm × 17.2 nm and a minimum distance to the periodic image of ~4.2 nm in each case (along the direction perpendicular to the membrane plane). The systems were solvated again, and KCl ions were added to each compartment separately, as described above. All systems were again energy-minimized and equilibrated for a further 1 ns. From the obtained simulation systems, data-acquisition runs were started, each lasting 200 ns, from which the first 10 ns were omitted from the analysis. Configurations were stored every 10 ps. Mean distances and mean thicknesses were calculated from the last 190 ns of each simulation run based on the distances between the average P-atom layers.

Leaflet-internal electrostatic potentials (see Supplementary Fig. 2c) were calculated as the time-average of the electrostatic interactions between all atoms within each leaflet. For the representation in Supplementary Fig. 2c, the electrostatic potentials of the two inner and outer leaflets were then averaged.

In order to determine the time sequence of headgroup tilt, area shrinkage and bilayer thickening, we carried out 500 independent non-equilibrium simulations, of the transition of two equilibrated single membranes in close contact to an equilibrated, i.e., thickened, double-membrane.

500 start structures were obtained by taking snapshots every 1 ns from a seeding trajectory of a single bilayer. The bilayers were then doubled as described above to obtain non-equilibrated double-membranes at a distance of 0.5 nm, and each replica was simulated without any biasing potential. A simulation time of 1 ns proved to be sufficient to identify the sequence of headgroup tilt, area shrinkage, and thickening (see Fig. 4f). To allow analysis of the equilibration process, trajectory coordinates were stored every 0.1 ps.

To determine the timescales of headgroup tilt, area, and thickness changes, exponential relaxations of the form $f\left(t\right)={x}_{\infty }+\left({x}_{0}-{x}_{\infty }\right){e}^{-\frac{t}{\tau }}$ were fitted to the data of each replica. Here, ${x}_{0}$ denotes the initial value of the observable (e.g., bilayer thickness at the beginning of the simulation), ${x}_{\infty }$ is the mean in the double-membrane equilibrium, and $\tau$ is the relaxation time. The values obtained for ${\tau }_{\text{tilt}}$, ${\tau }_{\text{area}}$, and ${\tau }_{\text{thickn}}$ yield the time sequence of headgroup tilt, area shrinkage, and bilayer thickening, as shown in Supplementary Fig. 2e, f.

Headgroup tilts, measured using the angle $\phi$ between N and P atoms of each lipid and the membrane normal (*z*-axis), were enforced by adding the angle-restraint term $k\left(1-{{\cos }}\left(\phi -{\phi }_{0}\right)\right)$ to the force field. To bias separated bilayers towards more tilted headgroups, the minimum of the potential ${\phi }_{0}$ was placed at 90°. Similarly, the potential minimum was placed at 0° to bias double bilayers at close distance (0.5 nm) towards less tilted head groups. In both cases, the force constant was varied between 0.5 and 30 kJ$\cdot$mol^−1^, depending on the desired biasing strength, and each simulation lasted 200 ns.

### Reporting summary

Further information on research design is available in the Nature Research Reporting Summary linked to this article.

## Supplementary information

Supplementary Information

Description of Additional Supplementary Files

Supplementary Movie 1

Supplementary Movie 2

Supplementary Movie 3

Supplementary Movie 4

Reporting Summary

## Data Availability

Data supporting the findings of this manuscript are available from the corresponding authors upon reasonable request. A reporting summary for this Article is available as a Supplementary Information file. Source data are provided with this paper.

## Source data

Source Data

## Code availability

Code used for cryoEM image analysis is available on Zenodo^24^. All molecular dynamics simulations were carried out with the publicly available GROMACS software (www.gromacs.org). Input parameter files, analysis scripts as well as the code to produce Supplementary Fig. 1 are available on Gitlab: https://gitlab.gwdg.de/lheinz/membrane-thickness-at-close-distance.

## References

[1] Hernández JM, Podbilewicz B (2017). The hallmarks of cell-cell fusion. Dev. Camb. Engl..

[2] Leikin SL, Kozlov MM, Chernomordik LV, Markin VS, Chizmadzhev YA (1987). Membrane fusion: overcoming of the hydration barrier and local restructuring. J. Theor. Biol..

[3] Risselada HJ, Kutzner C, Grubmüller H (2011). Caught in the act: visualization of SNARE-mediated fusion events in molecular detail. Chembiochem Eur. J. Chem. Biol..

[4] Kozlov MM, Chernomordik LV (2015). Membrane tension and membrane fusion. Curr. Opin. Struct. Biol..

[5] Jahn R, Fasshauer D (2012). Molecular machines governing exocytosis of synaptic vesicles. Nature.

[6] Rizo J, Xu J (2015). The synaptic vesicle release machinery. Annu. Rev. Biophys..

[7] Chang S, Trimbuch T, Rosenmund C (2018). Synaptotagmin-1 drives synchronous Ca2+-triggered fusion by C 2 B-domain-mediated synaptic-vesicle-membrane attachment. Nat. Neurosci..

[8] Hernandez JM (2012). Membrane fusion intermediates via directional and full assembly of the SNARE complex. Science.

[9] Yavuz H (2018). Arrest of trans-SNARE zippering uncovers loosely and tightly docked intermediates in membrane fusion. J. Biol. Chem..

[10] Imig C (2014). The morphological and molecular nature of synaptic vesicle priming at presynaptic active zones. Neuron.

[11] Brandt T, Cavellini L, Kühlbrandt W, Cohen MM (2016). A mitofusin-dependent docking ring complex triggers mitochondrial fusion in vitro. eLife.

[12] Gui L, Ebner JL, Mileant A, Williams JA, Lee KK (2016). Visualization and sequencing of membrane remodeling leading to influenza virus fusion. J. Virol..

[13] Shrestha BR, Banquy X (2016). Hydration forces at solid and fluid biointerfaces. Biointerphases.

[14] Pobbati AV, Stein A, Fasshauer D (2006). N- to C-terminal SNARE complex assembly promotes rapid membrane fusion. Science.

[15] Witkowska A, Spindler S, Mahmoodabadi RG, Sandoghdar V, Jahn R (2020). Differential diffusional properties in loose and tight docking prior to membrane fusion. Biophys. J.

[16] Kanduč M, Schneck E, Netz RR (2013). Hydration interaction between phospholipid membranes: insight into different measurement ensembles from atomistic molecular dynamics simulations. Langmuir.

[17] Wiederhold K, Fasshauer D (2009). Is assembly of the SNARE complex enough to fuel membrane fusion?. J. Biol. Chem..

[18] Schuette CG (2004). Determinants of liposome fusion mediated by synaptic SNARE proteins. Proc. Natl Acad. Sci. USA.

[19] Fasshauer D, Antonin W, Margittai M, Pabst S, Jahn R (1999). Mixed and non-cognate SNARE complexes. Characterization of assembly and biophysical properties. J. Biol. Chem..

[20] Siddiqui TJ (2007). Determinants of synaptobrevin regulation in membranes. Mol. Biol. Cell.

[21] Witkowska A, Jablonski L, Jahn R (2018). A convenient protocol for generating giant unilamellar vesicles containing SNARE proteins using electroformation. Sci. Rep..

[22] Schindelin J (2012). Fiji: an open-source platform for biological-image analysis. Nat. Methods.

[23] Eaton, J. W., Bateman, D., Hauberg, S. & Wehbring, R. *GNU Octave version 4.4.0 manual: a high-level interactive language for numerical computations* (2018).

[24] Witkowska, A. Measurement of membrane thickness in cryoEM images of liposomes. *Zenodo*10.5281/zenodo.4642359 (2021).

[25] Berendsen HJC, van der Spoel D, van Drunen R (1995). GROMACS: a message-passing parallel molecular dynamics implementation. Comput. Phys. Commun..

[26] Van Der Spoel D (2005). GROMACS: fast, flexible, and free. J. Comput. Chem..

[27] Hess B, Kutzner C, van der Spoel D, Lindahl E (2008). GROMACS 4: algorithms for highly efficient, load-balanced, and scalable molecular simulation. J. Chem. Theory Comput..

[28] Pronk S (2013). GROMACS 4.5: a high-throughput and highly parallel open source molecular simulation toolkit. Bioinformatics Oxf. Engl..

[29] MacKerell AD (1998). All-atom empirical potential for molecular modeling and dynamics studies of proteins. J. Phys. Chem. B.

[30] Huang J (2017). CHARMM36m: an improved force field for folded and intrinsically disordered proteins. Nat. Methods.

[31] Miyamoto S, Kollman PA (1992). Settle: an analytical version of the SHAKE and RATTLE algorithm for rigid water models. J. Comput. Chem..

[32] Hess B, Bekker H, Berendsen HJC, Fraaije JGEM (1997). LINCS: a linear constraint solver for molecular simulations. J. Comput. Chem..

[33] Darden T, York D, Pedersen L (1993). Particle mesh Ewald: An N⋅log(N) method for Ewald sums in large systems. J. Chem. Phys..

[34] Jones JE, Chapman S (1924). On the determination of molecular fields. —II. From the equation of state of a gas. Proc. R. Soc. Lond. Ser. Contain. Pap. Math. Phys. Character.

[35] Bussi G, Donadio D, Parrinello M (2007). Canonical sampling through velocity rescaling. J. Chem. Phys..

[36] Andersen HC (1980). Molecular dynamics simulations at constant pressure and/or temperature. J. Chem. Phys..

[37] Parrinello M, Rahman A (1981). Polymorphic transitions in single crystals: a new molecular dynamics method. J. Appl. Phys..

[38] Ghahremanpour MM, Arab SS, Aghazadeh SB, Zhang J, van der Spoel D (2014). MemBuilder: a web-based graphical interface to build heterogeneously mixed membrane bilayers for the GROMACS biomolecular simulation program. Bioinformatics.

[39] Stein A, Weber G, Wahl MC, Jahn R (2009). Helical extension of the neuronal SNARE complex into the membrane. Nature.

