# Supplementary material for: Tight docking of membranes before fusion represents a metastable state with unique properties
Source: Nat Commun. 2021 Jun 14;12:3606. doi: 10.1038/s41467-021-23722-8 (PMC8203622; doi:10.1038/s41467-021-23722-8)
Supplement: Supplementary file 1 — Supplementary Information [file 41467_2021_23722_MOESM1_ESM.pdf]

Supplementary Information for

Tight docking of membranes before fusion represents a metastable state with unique properties

Agata Witkowska[1,2,*], Leonard P. Heinz[3], Helmut Grubmüller[3], Reinhard Jahn[1,4,*]

[1] Laboratory of Neurobiology, Max-Planck-Institute for Biophysical Chemistry, Göttingen, Germany.

[2] Present address: Department of Molecular Pharmacology & Cell Biology, Leibniz-Forschungsinstitut für Molekulare Pharmakologie (FMP), Berlin, Germany.

[3] Department of Theoretical and Computational Biophysics, Max-Planck-Institute for Biophysical Chemistry, Göttingen, Germany.

[4] University of Göttingen, Göttingen, Germany.

*Correspondence to: rjahn@gwdg.de (R.J.), agata.witkowska@wp.eu (A.W.)

EM projections in membrane thickness analysis

In order to estimate to what extent the position of and distance between the peaks shown in Fig. 3a might be changed by projecting the curved spherical surface onto the two-dimensional image plane, we generated a three-dimensional density model for the vesicle with known geometry (Supplementary Figure 1 left) and carried out the same radial averaging analysis as in Fig 3a (shown in Supplementary Figure 1 right).

The model density was generated by superposition of two spherical shells for the two membrane leaflets, each with a Gaussian profile. The distances from the center of these Gaussian shells were chosen similar to the vesicle shown in Fig. 3a, 33 nm and 37 nm, respectively, with an inter leaflet distance of 4 nm as typical for the distance of the lipid head groups. The width of the Gaussians, 0.75 nm, was also chosen to resemble the radial density profiles in Fig 3a.

Figure S1 (right) shows this radial density distribution (thin lines) as well as the radially averaged projection on the image plane (thick line). As can be seen, the projection of the curved surface causes a small shift of the density maxima by ca. 0.1 nm towards smaller radii; however, this shift is very similar for the two density peaks, such that their mutual distance is essentially unaffected.

Monitoring the membrane curvature

To test for unwanted bending or buckling of the membranes during the simulations, which could potentially compromise the distance and thickness calculations, mean curvatures were calculated using the program g_lomepro [1] on a 5 × 5 grid for all four leaflets. In addition, we calculated the average phosphor layer spread as

$$\sigma_{tot} = \sqrt{\frac{1}{4}\sum_{l=1}^{4}\overline{\sigma_l^2(t)}},$$

where $\overline{\sigma_l^2(t)}$ denotes the time-averaged variance of the 100 phosphor atom positions in leaflet $l = \{1, 2, 3, 4\}$.

Supplementary Table 1. Absolute mean curvature averaged over all grid-points and all leaflets at various bilayer distances for the 100% DOPC-system.

Bilayer distance (nm)	Average absolute mean curvature (nm^{-1})	Average phosphor layer spread σ_{tot} (nm)
0.51	0.021	0.23
0.58	0.010	0.20
0.88	0.007	0.22
0.22	0.004	0.22
1.60	0.004	0.23
4.44	0.004	0.23

As shown in Supplementary Table 1, the mean membrane curvatures increase slightly for close bilayer distances as the membranes "roughen up" marginally but remain essentially flat (within the box size of approximately 8.1 nm) throughout the tested distance range. The average phosphor layer spread remains unaffected by the bilayer distance, further underscoring that no unwanted buckling of the membrane is seen. Note that contrary to σ_{tot}, the error bars in Fig. 4a were furthermore averaged over 100 phosphor atoms per leaflet and are therefore smaller by a factor of ~ 10.

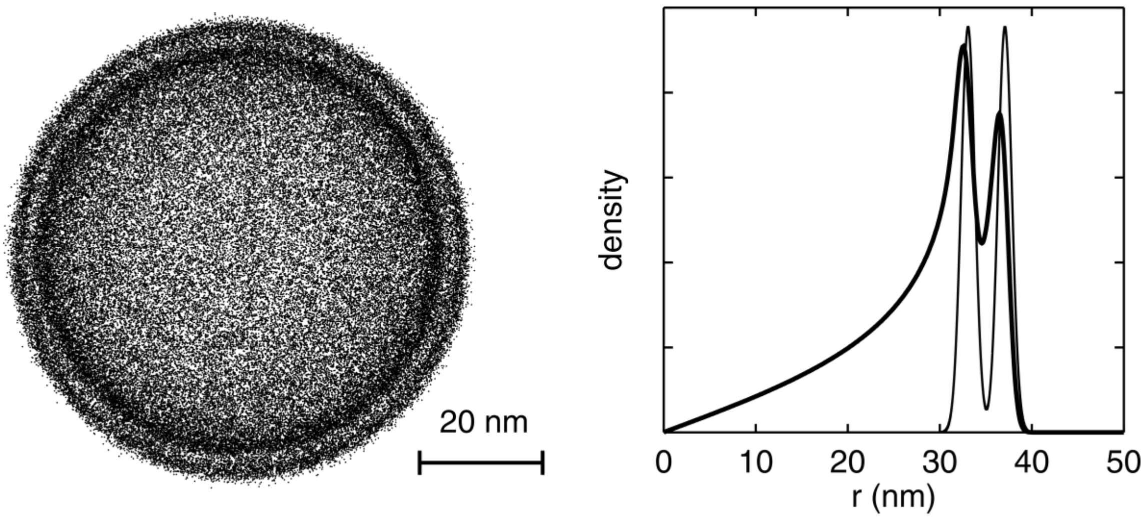

Supplementary Figure 1. Simulated EM projection of a spherical vesicle model density and radial density profiles. Left: Vesicle density model with 75 nm diameter, projected onto the image plane as in the EM images shown in Fig 3a. Right: Radial density distribution used to construct the vesicle density model (thin line); radial density distribution (thick line) obtained after radially averaging the two-dimensional projection on the left, as indicated in Fig. 3a. Source data are provided as a Source Data file.

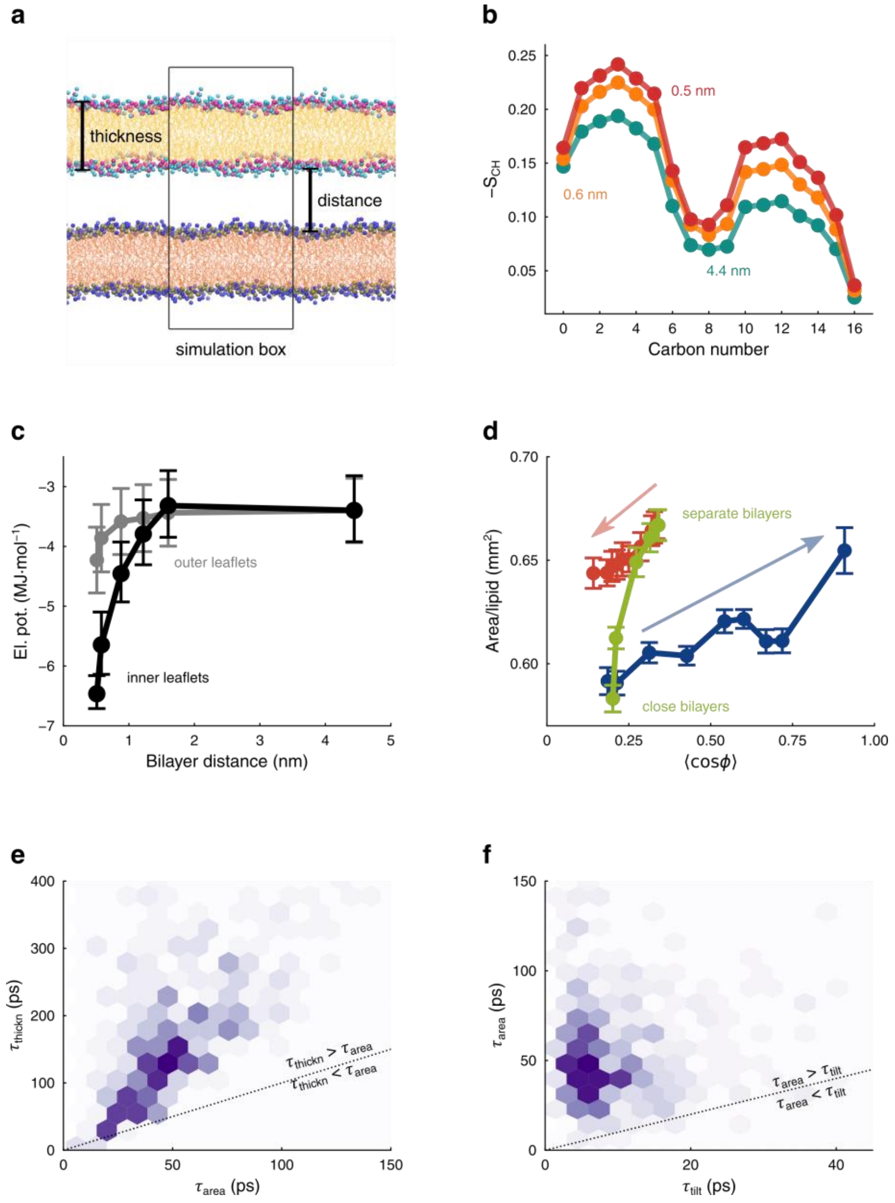

Supplementary Figure 2. Molecular dynamics simulations of membranes at close distances.

(**a**) Double-membrane simulation setup. The two bilayers are shown in yellow and orange; P-atoms are golden and pink, N-atoms are shown in blue and cyan color. (**b**) Deuterium order parameters for membrane consisting of 100% DOPC at selected distances. (**c**) Average leaflet-internal electrostatic potential for inner (blue) and outer (red) leaflets depending on the bilayer distance. (**d**) Relationship between headgroup tilt (cosϕ) and area per lipid. The data of membrane consisting of 100% DOPC at various distances is shown in green. Results from in-plane biased simulations of separated bilayers and out-of-plane biased simulations of bilayers at close distance are shown in red and blue, respectively. Data points (**c, d**) are averages from 19000 points (190 ns) ± SD as described in *Methods*. (**e**, **f**) Histograms of the response times of area shrinkage, bilayer thickening, and head group tilt, showing that the tilt precedes the area shrinkage, which precedes the thickening. Source data are provided as a Source Data file.

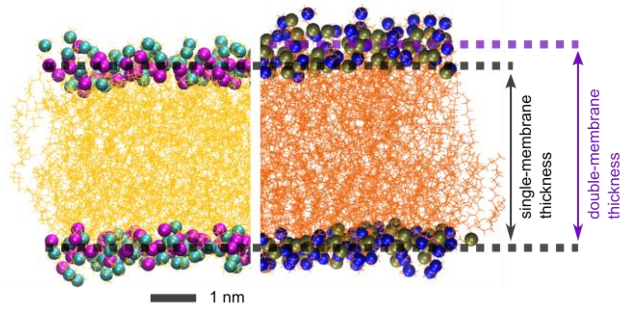

Supplementary Figure 3. Molecular dynamics snapshots illustrating the membrane thickening at close distances.

Snapshot of an equilibrated single-membrane (left) and an equilibrated double-membrane at 0.5 nm distance (right) consisting of 100% DOPC. For simplicity, only the top bilayer of the double-membrane setup is shown. P-atoms are golden and pink, N-atoms are shown in blue and cyan color; other atoms are shown in yellow and orange respectively.

References

1. Gapsys, V., de Groot, B. L. & Briones, R. Computational analysis of local membrane properties. *J Comput Aided Mol Des* **27**, 845–858 (2013).
