# Supplementary material for: Tight docking of membranes before fusion represents a metastable state with unique properties
Source: Nat Commun. 2021 Jun 14;12:3606. doi: 10.1038/s41467-021-23722-8 (PMC8203622; doi:10.1038/s41467-021-23722-8)
Supplement: Supplementary file 2 — Description of Additional Supplementary Files [file 41467_2021_23722_MOESM2_ESM.pdf]

Description of Additional Supplementary Files

File Name: **Supplementary Movie 1. Loose docking represents productive membrane fusion intermediate.**

Description: Time series of loosely docked GUVs transiting to the fully fused state. One GUV contains the membrane label DiD (magenta) and a Q-SNARE acceptor complex, and the second contains the OG labelled syb docking mutant (green). Movie displays approximately 1 min-long time series. First frame added from image acquired 1 min earlier. Same as in Fig. 2a.

File Name: **Supplementary Movie 2. Tight docking represents productive membrane fusion intermediate.**

Description: Time series of tightly docked GUVs transiting to the fully fused state. One GUV contains membrane label DiD (magenta) and a Q-SNARE acceptor complex, and the second contains OG labelled syb docking mutant (green). Movie displays 50 s-long time series. Same as in Fig. 2b.

File Name: **Supplementary Movie 3. Molecular dynamics simulation (190 ns) of the double-membrane setup (100% DOPC) at 0.58 nm.**
Description: Colors are identical to Supplementary Figures 2a and 3.

File Name: **Supplementary Movie 4. Tilted headgroups in the double-membrane system.**

Description: Twelve randomly selected headgroup dipole vectors from the bottom bilayer of a double-membrane system (100% DOPC at 0.58 nm distance). Headgroups of the top leaflet (magenta arrows) are in close contact with the top bilayer (not shown for clarity) and are tilted in-plane. Headgroups of the bottom leaflet (black arrows) are fully hydrated and are, on average, less tilted. Remaining colors are identical to Supplementary Figures 2a and 3.
