# Supplementary material for: Tight docking of membranes before fusion represents a metastable state with unique properties
Source: Nat Commun. 2021 Jun 14;12:3606. doi: 10.1038/s41467-021-23722-8 (PMC8203622; doi:10.1038/s41467-021-23722-8)
Supplement: Supplementary file 7 — Reporting Summary [file 41467_2021_23722_MOESM7_ESM.pdf]

nature research

Corresponding author(s): Reinhard Jahn, Agata Witkowska

Last updated by author(s): May 1, 2021

# Reporting Summary

Nature Research wishes to improve the reproducibility of the work that we publish. This form provides structure for consistency and transparency in reporting. For further information on Nature Research policies, see our Editorial Policies and the Editorial Policy Checklist.

## Statistics

For all statistical analyses, confirm that the following items are present in the figure legend, table legend, main text, or Methods section.

| n/a | Confirmed | |
|---|---|---|
| ☐ | ☒ | The exact sample size (*n*) for each experimental group/condition, given as a discrete number and unit of measurement |
| ☒ | ☐ | A statement on whether measurements were taken from distinct samples or whether the same sample was measured repeatedly |
| ☐ | ☒ | The statistical test(s) used AND whether they are one- or two-sided<br>*Only common tests should be described solely by name; describe more complex techniques in the Methods section.* |
| ☒ | ☐ | A description of all covariates tested |
| ☒ | ☐ | A description of any assumptions or corrections, such as tests of normality and adjustment for multiple comparisons |
| ☐ | ☒ | A full description of the statistical parameters including central tendency (e.g. means) or other basic estimates (e.g. regression coefficient) AND variation (e.g. standard deviation) or associated estimates of uncertainty (e.g. confidence intervals) |
| ☐ | ☒ | For null hypothesis testing, the test statistic (e.g. *F*, *t*, *r*) with confidence intervals, effect sizes, degrees of freedom and *P* value noted<br>*Give P values as exact values whenever suitable.* |
| ☒ | ☐ | For Bayesian analysis, information on the choice of priors and Markov chain Monte Carlo settings |
| ☒ | ☐ | For hierarchical and complex designs, identification of the appropriate level for tests and full reporting of outcomes |
| ☒ | ☐ | Estimates of effect sizes (e.g. Cohen's *d*, Pearson's *r*), indicating how they were calculated |

*Our web collection on statistics for biologists contains articles on many of the points above.*

## Software and code

Policy information about availability of computer code

| | |
|---|---|
| Data collection | Confocal microscopy data were collected with ZEN black 2012 software. |
| Data analysis | Fiji (with ImageJ 1.52p) and GNU Octave (5.1.0) custom code was used to analyse cryoEM data. Code was deposited on Zenodo (doi.org/10.5281/zenodo.4642359). 3D reconstructions were prepared with Imaris 9.2 (Bitplane). All molecular dynamics simulations were carried out with the publicly available GROMACS software (www.gromacs.org). Input parameter files, analysis scripts as well as the code to produce Supplementary Figure 1 are available on Gitlab: https://gitlab.gwdg.de/lheinz/membrane-thickness-at-close-distance. |

For manuscripts utilizing custom algorithms or software that are central to the research but not yet described in published literature, software must be made available to editors and reviewers. We strongly encourage code deposition in a community repository (e.g. GitHub). See the Nature Research guidelines for submitting code & software for further information.

## Data

Policy information about availability of data

All manuscripts must include a data availability statement. This statement should provide the following information, where applicable:

- Accession codes, unique identifiers, or web links for publicly available datasets
- A list of figures that have associated raw data
- A description of any restrictions on data availability

The authors declare that all data supporting the findings of this study are available within the article and its supplementary information files or from corresponding authors upon reasonable request.

# Field-specific reporting

Please select the one below that is the best fit for your research. If you are not sure, read the appropriate sections before making your selection.

☒ Life sciences ☐ Behavioural & social sciences ☐ Ecological, evolutionary & environmental sciences

For a reference copy of the document with all sections, see nature.com/documents/nr-reporting-summary-flat.pdf

# Life sciences study design

All studies must disclose on these points even when the disclosure is negative.

| | |
|---|---|
| Sample size | No statistical methods were used to predetermine sample size. Sample size was limited by the throughput as only limited number of events occured in each attempt. For each experimental figure experiments were replicated multiple times and pooled together for analysis. Minimum of 3 independent experiments were performed. |
| Data exclusions | Exclusion criteria were pre-established: GUVs pairs that appeared as docked but contained membrane dye in both vesicles were excluded from the analysis. |
| Replication | All attempts of replication of loose/tight docking morphology were successful. For each experimental figure experiments were replicated multiple times and pooled together for analysis. For each condition, minimum of 3 independent experiments were performed. |
| Randomization | n/a There was no experimental group allocation. |
| Blinding | n/a There was no experimental group allocation. |

# Reporting for specific materials, systems and methods

We require information from authors about some types of materials, experimental systems and methods used in many studies. Here, indicate whether each material, system or method listed is relevant to your study. If you are not sure if a list item applies to your research, read the appropriate section before selecting a response.

## Materials & experimental systems

| n/a | Involved in the study | |
|---|---|---|
| ☒ | ☐ | Antibodies |
| ☒ | ☐ | Eukaryotic cell lines |
| ☒ | ☐ | Palaeontology and archaeology |
| ☒ | ☐ | Animals and other organisms |
| ☒ | ☐ | Human research participants |
| ☒ | ☐ | Clinical data |
| ☒ | ☐ | Dual use research of concern |

## Methods

| n/a | Involved in the study | |
|---|---|---|
| ☒ | ☐ | ChIP-seq |
| ☒ | ☐ | Flow cytometry |
| ☒ | ☐ | MRI-based neuroimaging |
